# Differences in energy and nutritional content of menu items served by popular UK chain restaurants with versus without voluntary menu labelling: A cross-sectional study

**DOI:** 10.1371/journal.pone.0222773

**Published:** 2019-10-16

**Authors:** Dolly R. Z. Theis, Jean Adams

**Affiliations:** Centre for Diet and Activity Research, MRC Epidemiology Unit, University of Cambridge, Cambridge, United Kingdom; University of Florida, UNITED STATES

## Abstract

**Background:**

Poor diet is a leading driver of obesity and morbidity. One possible contributor is increased consumption of foods from out of home establishments, which tend to be high in energy density and portion size. A number of out of home establishments voluntarily provide consumers with nutritional information through menu labelling. The aim of this study was to determine whether there are differences in the energy and nutritional content of menu items served by popular UK restaurants with versus without voluntary menu labelling.

**Methods and findings:**

We identified the 100 most popular UK restaurant chains by sales and searched their websites for energy and nutritional information on items served in March-April 2018. We established whether or not restaurants provided voluntary menu labelling by telephoning head offices, visiting outlets and sourcing up-to-date copies of menus. We used linear regression to compare the energy content of menu items served by restaurants with versus without menu labelling, adjusting for clustering at the restaurant level. Of 100 restaurants, 42 provided some form of energy and nutritional information online. Of these, 13 (31%) voluntarily provided menu labelling. A total of 10,782 menu items were identified, of which total energy and nutritional information was available for 9605 (89%). Items from restaurants with menu labelling had 45% less fat (beta coefficient 0.55; 95% CI 0.32 to 0.96) and 60% less salt (beta coefficient 0.40; 95% CI 0.18 to 0.92). The data were cross-sectional, so the direction of causation could not be determined.

**Conclusion:**

Menu labelling is associated with serving items with less fat and salt in popular UK chain restaurants. Mandatory menu labelling may encourage reformulation of items served by restaurants. This could lead to public health benefits.

## Introduction

Globally, obesity has almost tripled since 1975, making it one of the most pressing public health challenges today[1]. Poor diet is a leading contributor to obesity, morbidity and mortality internationally[2]. Food from out of home sources, such as restaurants and fast food takeaways, tends to be high in energy, fat, sugar and salt[3–6]. Frequent consumption of food prepared out of the home is associated with poorer dietary quality and increased body weight[7–9].

One commonly proposed intervention to improve the nutritional quality of foods served by and selected from out of home food outlets is menu labelling. Typically menu labelling involves making nutritional information on foods served by out of home establishments available at the point of order or purchase[10]. Mandatory menu labelling in large chains was introduced in the US in May 2018 and has been implemented in some parts of Australia since 2012[11]. In the UK, voluntary menu labelling was included in the government’s Public Health Responsibility Deal in 2011[12]. Mandatory menu labelling was proposed in the second chapter of the government’s Childhood Obesity Plan in summer 2018[13], and a consultation to inform how such a policy might be implemented was launched in September 2018[14].

Menu labelling is typically conceived of as an information-giving intervention. In this framing, the assumption is that providing customers with clearer information on the energy and nutritional content of food served will allow them to make more informed, and hence ‘better’, choices. This conceptualisation of menu labelling is as a high agency intervention that relies on individuals using substantial personal resources to benefit from the intervention. Numerous systematic reviews, including a recent Cochrane review[15], have found only modest, poor quality, evidence of an effect of menu labelling on customer purchasing and consumption[15–25].

It is also possible that menu labelling acts as a low agency intervention by changing what outlets serve. In this framing, outlets are considered to perceive public information on excessively high energy (and other nutrient) content to equate to bad publicity and engage in reformulation, or development of new ‘healthier’ products, before implementing menu labelling. Reformulation is then expected to lead to changes in what consumers eat, without necessarily requiring that they use the information provided to inform changes in what they purchase.

Evidence on whether menu labelling affects the content of menu items served by restaurants is mixed. One 2018 meta-analysis by Zlatevska et al found that, on average, retailers reduced the energy content of items they serve by 15kcal after implementation of menu labelling[26]. However, substantial data included in this meta-analysis were collected in the context of known impending mandatory menu labelling, or implementation of such mandation. This may have substantially impacted the results found. No data from the UK were included. One 2017 systematic review by Bleich et al of five studies examining changes in the content of US restaurant menu items offered following implementation of local menu labelling regulations or in advance of national implementation found mixed effects. Two of the included studies found no statistically significant difference and three found a statistically significant difference in energy content[19].

We aimed to determine whether there were differences in the energy and nutritional content of menu items served by popular UK chain restaurants with versus without voluntary menu labelling. Our data was collected before government proposals for mandatory menu labelling had been published meaning they were uncontaminated by any retailer preparation for implementation of such an intervention.

## Methods

We sourced nutritional information on menu items served by large UK chain restaurants from restaurant websites; and used this to compare information from chains that did and did not voluntarily provide menu labelling.

### Restaurant inclusion criteria

Popular chain restaurants were defined as those listed in Technomic’s (a foodservice consultancy) “Top 100 U.K. Chain Restaurants Ranking” which were ranked by their total UK foodservice sales in 2013[27]. The list contains different types of restaurants including those with predominantly dine-in facilities and those with predominantly takeaway facilities. Henceforth, we refer to all included establishments as restaurants. Restaurants on this list were included in the analysis if they provided online nutritional information on food served in the restaurants.

### Menu item inclusion criteria

Nutritional data were sourced from included restaurants’ websites in March-April 2018. Where a restaurant chain had different menus for use in different outlets (e.g. a number of pub chains provided ‘Core’, ‘Urban’ and ‘Rural’ menus) the most mainstream menu, used in the highest proportion of outlets, was used. Data were collected for all items on included menus as they appeared on websites. The only exception was when there was a negative value presented for energy or any nutrient. As negative values are implausible, these were regarded as errors and so were recorded as missing.

Where multiple menu items with identical item names featured on the same menu, each item was included because nutritional information occasionally varied–perhaps due to portion size variations. Beverages with options for different types of milk (e.g. cappuccino made with coconut milk and cappuccino made with semi skimmed milk) were entered individually so comparisons could be made between the different types. In two instances, platters composed of individual items served together were excluded as there was no nutritional information available for them, but the individual component items for which nutritional information was available were included.

There was some inconsistency in portion sizes of pizzas–particularly where these were intended for more than one person. We used energy and nutritional information on whole pizzas where these were intended for one person, and on three slices where it was clearly stated on menus that pizzas were intended to be shared.

### Nutritional data

Data collected exactly as shown on included restaurants’ websites included: restaurant name, menu item name, and total energy and nutritional content of all included menu items. Alongside total energy, information on the following nutrients was extracted where available: total fat, total saturated fat, total carbohydrates, total sugar, total fibre, total protein and total salt.

### Menu item categorisation

Menu item names were used to identify whether each item was labelled as shareable or not, for example John Barras’ “House Sharing Platter”, to separate items presented as being for sharing from items presented as being for an individual. Item descriptions were also used to categorise all items into one of 12 food categories, derived from similar work in the US[28] (Table 1).

**Table 1:** Food categories and descriptions.

Food category	Description and examples
Appetisers & Sides	Items designed to supplement a main course e.g. chicken wings, sides of vegetables, rice, beans, fruit portion, coleslaw, potato salad, dumplings, nachos (irrespective of where they appear on the menu).
Baked Goods	Foods prepared with flour, baked, and served on their own, e.g. breads and rolls, muffins, doughnuts, croissants.
Beverages	All drinks including ice cream smoothies and milk shakes e.g. sugary and sweetened carbonated beverages, juice, milk, coffee, tea, smoothies, hot chocolate, beer, wine, milkshakes, floats, frappes.
Burgers	All items described as burgers e.g. hamburger, cheeseburger, chicken burger, veggie burgers.
Desserts	All sweets, including baked goods served as a dessert e.g. ice cream, cakes, cupcakes, brownies, cookies, pies, cheesecake, dessert bars, frozen yoghurt, sundaes. Excludes milkshakes.
Fried Potatoes	French fries (chips), sweet potato fries, fried potato skins e.g. potato wedges, hash browns, loaded fries which are fries with toppings. Excludes mashed or baked potatoes.
Mains	A main course meal and multiple component main course meals e.g. chicken nuggets, pasta mains, rice bowls, waffles, French toast, pancakes, porridge, quiches, sushi, mac and cheese. Excludes items described as or in the menu section indicating appetisers & sides
Pizza	Any dish consisting of a flat base of dough with a range of toppings, often featuring cheese and tomato sauce. Includes flatbread.
Salads	Any cold dish of various mixtures of raw and cooked vegetables and salad leaves, including side salads and salads served with additional items e.g. chicken or steak. Excludes potato and pasta salads.
Sandwiches	Any sandwich items in bread or tortilla e.g. wraps, breakfast sandwiches, hot dogs, bagels. Sandwiches served in buffets in quarter portions were considered Appetisers & Sides.
Soup	Any liquid dishes with meat, vegetables, legumes, e.g. soups and stews, gumbo and chowders.
Toppings & Ingredients	Toppings and ingredients in build-your-own products or products described as ‘Add Ons’ or ‘Extras’ e.g. sauces, butter and spreads, salad dressing, salad bar items, beverage toppings such as whipped cream.

### Menu labelling

Information on whether restaurants had voluntary menu labelling was obtained by telephoning the head offices of each chain restaurant in May 2018. This was verified either by visiting one outlet from each chain in person or, where this was not possible, sourcing an up-to-date image of the menu online.

### Statistical analyses

The unit of analysis was the menu item, clustered within restaurants. Analyses were restricted to non-sharable items and those for which full data on total energy, fat, saturated fat, carbohydrate, sugar, protein and salt data was available. Fibre was excluded from the analyses due to 53% of data being missing. In some cases, stated macronutrient content was inconsistent with the stated energy content. The difference between stated energy content and expected energy content (calculated using stated fat, carbohydrate and protein) was determined and all menu items with more than +/-20% difference between expected and stated energy content were excluded from the analyses. We used +/-20% tolerance as this is the tolerance acceptable under current EU guidance on nutritional labelling on food packaging[29]. As nutritional variables were not normally distributed, non-parametric descriptive methods were used to summarise the data.

Separate linear regression models were used to compare log transformed energy and nutritional content of items from restaurants which did and did not voluntarily provide menu labelling. Variables were log transformed for analysis and regression coefficients back transformed for interpretation. Standard errors (and 95% confidence intervals) were adjusted to account for clustering at the restaurant level.

## Results

Forty-two restaurants published nutritional information on their websites and were included in the analysis. Of the remaining 58, two no longer existed at the time of data collection, one had a non-functioning website and the remaining 55 did not publish nutritional information online.

Table 2 shows the 100 restaurants considered for inclusion, ranked by 2013 UK sales (from Technomic’s list), and indicating whether each voluntarily published nutritional information online or provided menu labelling. Of the 42 included restaurants with online nutritional information, 13 (31%) voluntarily provided menu labelling. Eleven of the 13 restaurants that provided voluntary menu labelling were in the top 50 by sales in 2013; 33 of the 52 functioning restaurants with functioning websites that voluntarily provided neither menu labelling or online nutritional information were in the bottom 50 by sales in 2013.

**Table 2:** Total UK sales and UK units in 2013, presence of online nutritional information, and voluntary menu labelling in 100 popular UK chain restaurants.

Rank	Restaurant Name	2013 UK Sales (£000)*	2013 UK Units*	Online energy/nutritional information	Voluntary menu labelling
1	McDonald’s	£1,810,000	1,222	Yes	Yes
2	Wetherspoon	1,217,000	905	Yes	Yes
3	Costa Coffee	937,000	1,755	Yes	Yes
4	Greggs	787,000	1,671	Yes	Yes
5	KFC	684,500	850	Yes	Yes
6	Domino’s Pizza	622,500	771	Yes	No
7	Starbucks	606,000	764	Yes	Yes
8	Pizza Hut	532,000	685	Yes	No
9	Subway	531,000	1,590	Yes	Yes
10	Nando’s	455,000	290	Yes	No
11	PizzaExpress	411,000	421	Yes	No
12	Burger King	383,000	484	Yes	Yes
13	Pret A Manger	319,000	270	Yes	Yes (food only)
14	Vintage Inns	307,000	193	No	No
15	Caffe Nero	305,000	550	Yes	Yes (food only)
16	Frankie & Benny’s	207,000	209	No	No
17	Harvester Salad & Grill	196,000	210	No	No
18	Wagamama	179,000	105	Yes	No
19	Sizzling Pubs	174,000	220	No	No
20	Ember Inns	170,000	130	No	No
21	Brewers Fayre	163,000	145	Yes	No
22	Hungry Horse	161,000	199	No	No
23	T.G.I Friday’s	153,000	65	No	No
24	Beefeater Grill	146,000	140	Yes	No
25	Prezzo	136,000	194	No	No
26	Chef & Brewer Pub Co.	125,000	135	Yes	No
27	Crown Carveries	123,000	114	No	No
28	Table Table	116,000	105	Yes	No
29	Taylor Walker	112,000	113	Yes	No
30	Toby Carvery	112,000	154	Yes	No
31	Revolution Vodka Bars	109,000	67	No	No
32	Zizzi	109,000	130	Yes	No
33	Carluccio’s	104,000	76	No	No
34	Jamie’s Italian	102,000	37	Yes	No
35	EAT	99,000	112	Yes	Yes (food only)
36	Nicholson’s	99,000	77	No	No
37	ASK	95,000	110	Yes	No
38	Fayre & Square	95,000	157	Yes	No
39	The Slug and Lettuce	95,000	73	No	No
40	Café Rouge	87,000	127	No	No
41	Papa John’s	86,000	246	Yes	No
42	Yate’s	84,000	69	Yes	No
43	Sayers the Better Bakers	78,000	178	No	No
44	YO! Sushi	75,000	66	Yes	Yes
45	All Bar One	73,000	47	Yes	No
46	Ben & Jerry’s	72,000	265	Yes	No
47	Bella Italia	66,000	91	No	No
48	Strada	63,500	68	No	No
49	Chicken Cottage	61,000	129	Yes	No
50	John Barras	60,200	126	Yes	No
51	Chiquito	54,000	70	No	No
52	Gaucho Grill	53,200	16	No	No
53	Patisserie Valerie	53,000	108	No	No
54	Old English Inns	52,500	55	Yes	No
55	O’Neill’s	49,000	49	No	No
56	Scream	48,600	43	No longer exists	NA
57	Gourmet Burger Kitchen	46,200	60	Yes	No
58	Davy’s	45,100	28	No	No
59	Flaming Grill Pub Co.	42,000	87	Yes	No
60	Loch Fyne	42,000	42	No	No
61	Browns Bar & Brasserie	38,500	27	No	No
62	Giraffe	38,200	50	No	No
63	Brasserie Blanc	38,000	19	No	No
64	La Tasca	37,000	38	No	No
65	Cote Restaurants	36,700	46	No	No
66	Miller & Carter	35,000	29	No	No
67	Wildwood Restaurants	33,100	18	No	No
68	Wacky Warehouse	32,200	75	No	No
69	Hollywood Bowl	32,000	46	No	No
70	Favourite Fried Chicken	31,800	85	No	No
71	Pitcher & Piano	31,700	18	No	No
72	Byron	31,000	34	No	No
73	Meet & Eat Pub & Grill	31,000	38	No	No
74	Piccolino Ristorante e Bar	30,500	21	No	No
75	PAUL	30,400	31	Yes	No
76	Le Pain Quotidien	30,000	24	No	No
77	Las Iguanas	29,800	34	No	No
78	Little Chef	29,500	78	No	No
79	Loungers	29,500	38	No longer exists	NA
80	Cosmo	28,100	15	No	No
81	Handmade Burger Co.	27,300	18	No	No
82	San Carlo	27,000	13	No	No
83	Jamies Wine Bars	26,000	10	No	No
84	Wimpy	26,000	110	Yes	Yes
85	Ed’s Easy Diner	25,700	23	No	No
86	Pizza GoGo	25,700	95	No	No
87	Krispy Kreme	25,400	52	Yes	No
88	Bill’s	25,200	31	Yes	No
89	Busaba Eathai	25,200	10	No	No
90	Pizza Kitchen & Bar	25,200	24	Website invalid	No
91	Gusto	24,800	10	No	No
92	Muffin Break	23,300	51	No	No
93	Walkabout	23,200	27	Yes	No
94	Baguette Express	23,000	70	No	No
95	Chimichanga	22,800	37	No	No
96	AMT Coffee Bars	22,600	60	No	No
97	Dixy Chicken	22,300	82	No	No
98	Itsu	21,700	43	Yes	Yes
99	The Restaurant Bar & Grill	21,500	11	No	No
100	Aagrah	21,400	16	No	No

Green: restaurants with nutritional information available online and voluntary menu labelling

Orange: restaurants with nutritional information available online, but no voluntary menu labelling

Red: restaurants with no nutritional information available online, and no voluntary menu labelling

Unshaded: restaurant no longer existed at the time of data collection

* Based on Technomic’s 2013 list

Of 10,782 menu items identified across the 42 included restaurants, a total of 9,984 (93%) menu items with no missing data for energy, fat, saturated fat, carbohydrates, sugar, protein and salt were included in the analysis (see Table 3). Of these, 379 (4%) menu items were excluded for having more than +/-20% uncertainty of measurement. Of the remaining 9,605 menu items 6,811 (71%) were food items, 1,929 (20%) were beverages, and 865 (9%) were toppings or ingredients. Table 4 summarises the distribution of total content of energy and each nutrient per menu item across all included items. Daily Reference Intakes (DRIs) for each nutrient are also provided[30]. Across all menu categories, at least 75% of individual menu items were below DRIs for all nutrients. However, the maximum values for energy and each nutrient exceeded DRIs in all cases meaning that individual items were exceeding the entire daily recommended intake. The maximum values show that some individual items contained more than two times the daily recommended amount for energy, fat, saturated fat, sugar, protein or salt. For energy, the maximum value was 5961 meaning an individual menu item contained almost three times the daily recommended amount.

**Table 3:** Summary of non-missing data on energy and nutritional content of 10,782 menu items.

Energy/nutrient per serving	Complete Data, n(%)
Kcal	10,653 (99)
Fat (g)	10,535 (98)
Saturated Fat (g)	10,533 (98)
Carbohydrates (g)	10,333 (96)
Sugar (g)	10,538 (98)
Fibre (g)	5,097 (47)
Protein (g)	10,323 (96)
Salt (g)	10,447 (97)

**Table 4:** Distribution of energy and nutrients in 9605 menu items from 42 popular UK chain restaurants.

Energy/nutrient	Median (25^th^– 75^th^ centile)	Minimum—maximum	Daily reference intake
Energy (kcal)	327 (150–581)	1–5961	2000
Fat (g)	13.8 (5.2–25.5)	0–412	<70g
Saturated Fat (g)	4.8 (1.5–9.8)	0–162.2	<20g
Carbohydrates (g)	37.6 (15–63)	0–424.7	At least 260g
Sugar (g)	9.8 (3.6–18.5)	0–228.3	90g
Protein (g)	9.7 (3.5–26.7)	0–212.6	50g
Salt (g)	0.9 (0.21–2.7)	0–29	6g

The distribution of energy and nutrients in menu items, stratified by food category, is shown in S1 Table. In four categories (burgers, desserts, mains, and sandwiches) the maximum energy and nutritional content of items exceeded DRIs for five or six of the six variables considered.

**Fig 1:**
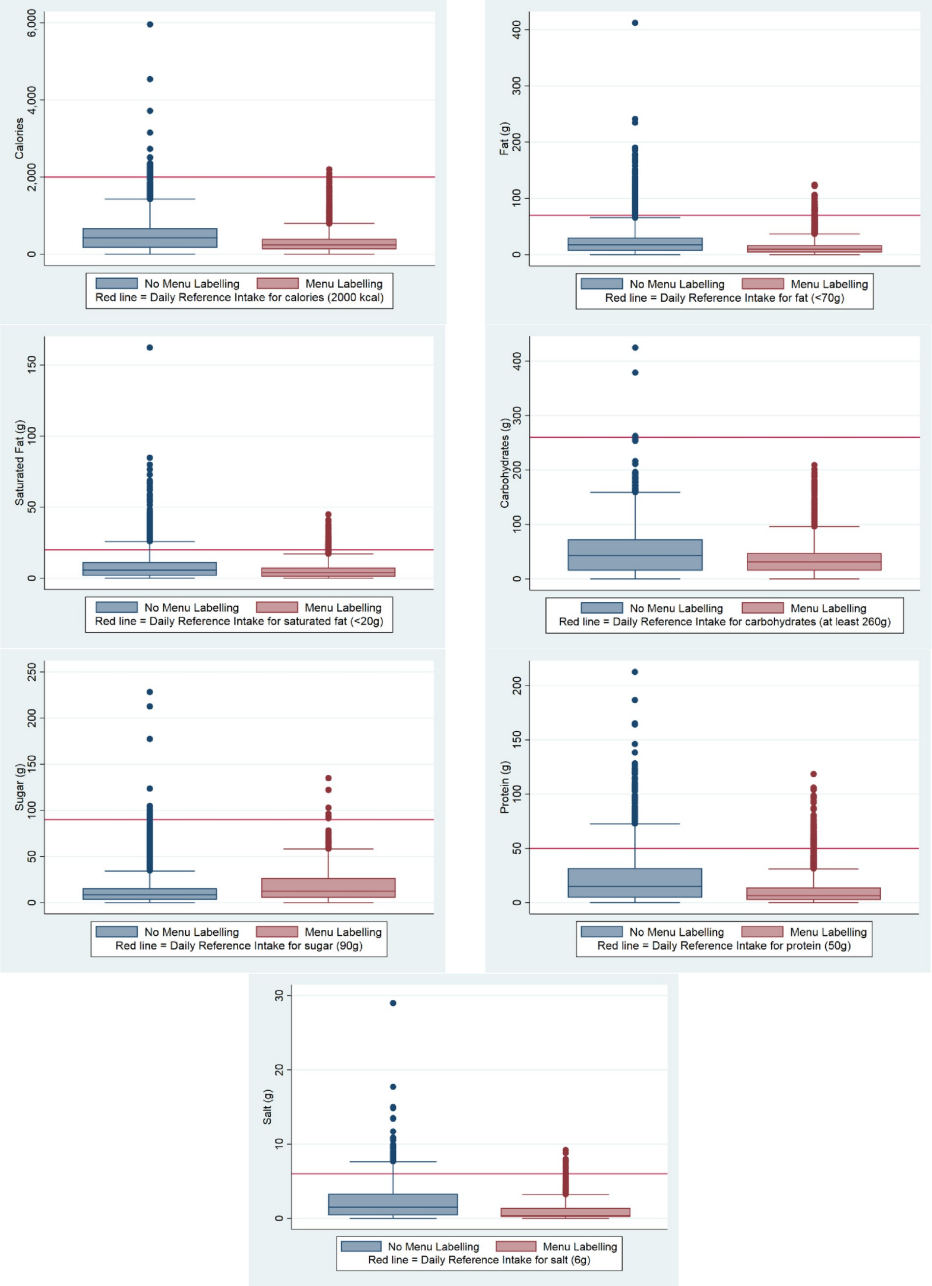
Distribution of energy and nutrients in 9605 menu items from 42 popular UK chain restaurants, stratified by whether or not restaurants provided voluntary menu labelling.

Fig 1 shows the distribution of energy and nutrients in individual menu items stratified by whether restaurants provided voluntary menu labelling or not. Medians for all variables, except sugar, were lower in items from restaurants with, compared to without, menu labelling. However, in all cases, except total carbohydrates, maximum values for items in both groups exceeded relevant DRIs.

Table 5 shows the exponentiated results of the separate linear regression models comparing log transformed energy and nutrients of menu items served by restaurants with and without voluntary menu labelling. The exponentiated coefficients are ratios of the geometric mean of each variable in restaurants with versus without menu labelling. A value less than 1 indicates the variable is lower in restaurants with menu labelling compared to those without. 95% confidence intervals that do not cross 1 indicate statistical significance. After adjusting for clustering at the restaurant level, fat and salt were significantly lower in items from restaurants with, versus without, voluntary menu labelling. Items from restaurants with menu labelling had 45% less fat and 60% less salt than those from restaurants without menu labelling. Although items from restaurants with menu labelling had 32% less energy, 35% less saturated fat, 17% less carbohydrates, 52% more sugar and 48% less protein than those from restaurants without menu labelling, the results were not statistically significant.

**Table 5:** Summary of linear regression models comparing log transformed energy and nutritional content of 9605 menu items from 42 popular UK restaurants with and without voluntary menu labelling.

Energy/nutrient	Exponentiated regression coefficient*	95% CI (adjusted for clustering at restaurant level)
Energy (kcal)	0.68	0.43 to 1.07
Fat (g)	0.55	0.32 to 0.96
Saturated Fat (g)	0.65	0.41 to 1.01
Carbohydrates (g)	0.83	0.54 to 1.28
Sugar (g)	1.52	0.91 to 2.54
Protein (g)	0.52	0.26 to 1.03
Salt (g)	0.40	0.18 to 0.92

*The ratio of the geometric mean of each variable in restaurants with versus without menu labelling.

Similar data to Table 5 stratified by food category is shown in S2 Table. The results are mixed and most differences in energy and nutrient content are not statistically significant. Some notable exceptions were that Baked Goods items from restaurants with menu labelling had, on average, 18% more energy, 74% more fat, 100% more saturated fat, 300% more sugar but 25% more protein and 43% more salt than items from restaurants without menu labelling; pizza items had 39% less sugar and 64% less salt; sandwich items had 39% less sugar, 23% less protein, and 27% less salt; and toppings & ingredients had 47% less fat, 44% less saturated fat, and 59% less protein than items from restaurants without menu labelling. No statistically significant differences was found in the energy or any nutrient contents of Appetisers & Sides, Beverages, Burgers, Desserts, Fried Potatoes, Mains, Salads and Soup items between restaurants with and without menu labelling.

## Discussion

This is the first assessment of differences in energy and nutritional content of menu items served by UK restaurants that do and do not provide voluntary menu labelling in a context where mandatory labelling had not been proposed or implemented. Two months before the UK government announced proposals for mandatory menu labelling, menu items served by popular UK restaurants with voluntary menu labelling had 45% less fat and 60% less salt than those from restaurants without menu labelling.

This is the first comprehensive survey of the energy and nutritional content of items served by popular UK chain restaurants, and the prevalence of providing information on these variables online and in-restaurant. Of 100 restaurant chains considered, 42 provided energy and nutritional information online, of which 13 provided any of this information on the restaurant menu. There were examples of single items that contained more than the DRI for energy and all nutrients considered from both restaurants that did and did not provide voluntary menu labelling.

### Strengths and weaknesses of methods

We used a cross-sectional design and reverse causation cannot be excluded. It is possible that restaurants serving menu items with less fat and salt are more likely to voluntarily menu label. However, in the absence of mandatory menu labelling, this would also be limitation of a longitudinal design.

We considered all chain restaurants in the top 100 by UK sales in 2013. This increases the generalisability of the findings to the UK chain sector in particular. However, the findings may be less generalizable to the independent sector, and to settings beyond the UK. We were unable to source more recent data on the top UK chain restaurants by sales. It is likely the chains on this list have changed somewhat since 2013.

Without laboratory analysis, we cannot confirm the validity or reliability of the information on energy or nutritional content used. Whilst some macronutrient values were inconsistent with stated total energy content, we do not know which values were erroneous–it could be that the macronutrient values were wrong, or that the total energy content was wrong. As such, we excluded items where stated energy content was +/-20% difference from energy calculated from macronutrients. The other values removed were negative values which are clearly implausible. Laboratory analysis was not feasible within the resources available to us. Nor did we have resources for duplicate transcription. Previous research indicates that in-restaurant data on energy content tends to be accurate overall[31].

Our statistical analysis was conducted at the menu item level. Different restaurants report meals and their component parts differently meaning that items are not necessarily comparable. Repeating our analyses stratified by food category overcame this limitation to some extent.

Our data describe menu items available for purchase. We do not know the relative frequency with which items are purchased and cannot determine the potential impact of menu labelling on purchasing or consumption.

### Interpretation of findings

We found lower fat and salt in items served by chains with, versus without, voluntary menu labelling, but no effect on energy content. Previous research comparing food content has largely focused on changes in energy content associated with menu labelling[11,18,26]. The majority of results, including from a meta-analysis, find that menu labelling is associated with healthful changes in the energy content of menu items. However, most previous studies which report an effect on energy content were reported in contexts where nation-wide mandatory menu labelling was implemented. Given we found no difference in energy content, such nation-wide mandatory labelling may be required to achieve significant change in energy content.

This study contributes to the evidence base in two key ways. As far as we understand, it is the first study to present differences in energy content as well as in fat, saturated fat, carbohydrates, sugar, protein and salt; and it is the first major UK study of differences in the energy and nutritional content of menu items served in restaurants with and without menu labelling. We found that the difference in energy and nutrient content between restaurants that did and did not have menu labelling were not consistent. Most previous studies in this area have focused on energy. Our findings that any impacts on energy are not generalisable to other nutrients indicate that future research should include a wider range of nutritional information than just energy.

It is difficult to determine the direction of any causation using cross-sectional data. However, this may not be an either/or situation. It is possible that menu labelling encourages change in the content of food served and simultaneously that those chains with ‘healthier’ offerings are more likely to label. Further research is required to determine why some restaurants opt for voluntary menu labelling. In the UK the Public Health Responsibility Deal[32] encouraged some restaurants to voluntarily menu label. It is notable that 11 of the 13 restaurants that provided voluntary menu labelling were in the top 50 by sales. Larger chains may come under more scrutiny from governments, the media, campaign groups and the public to provide both menu labelling and ‘healthier’ options.

We found inconsistent magnitudes of difference in energy and nutrients between chains that did and did not provide voluntary menu labelling. This suggests that menu labelling is not simply associated with reduced portion size (where energy and other nutrients would be reduced in comparable proportions). Rather, the formulation of items served by the two groups of restaurants appears to be different. Further work could explore these differences in more depth.

While restaurant characteristics may well be one factor influencing whether or not they choose to menu label, it would be difficult and potentially misleading to develop a comprehensive categorisation of these as many restaurants have multiple characteristics. For example, many restaurants provide both dine-in and takeaway facilities. Furthermore, dine-in experiences, for example, may differ between restaurants. The growth of online restaurant ordering platforms such as Deliveroo compound this problem.

We found individual items that substantially exceeded DRIs for energy and all nutrients studied. The highest energy content of a single menu item was almost three times the recommended daily energy intake for a UK adult. Similarly, individual items provided almost six times the DRI for fat, more than eight times the DRI for saturated fat, more than three times the DRI for sugar, and almost five times the DRI for salt. This indicates some exceedingly large portion sizes and nutritionally imbalanced items. Given that portion size is associated with consumption[33], this is likely to contribution to over-consumption at individual sittings. More than one quarter of UK adults eat meals out at least once a week[34], indicating that these large, nutritionally imbalanced portions are likely to contribute to poor dietary intake at a population level[35]. Recent efforts to encourage reduction of portion size across both the supermarket and out of home sectors in England may help address this in due course[36].

### Implications for policy, practice and research

Our findings indicate that mandatory menu labelling may lead to reformulation of existing items, or systematic changes in the content of newly introduced dishes. Alongside modest changes in purchasing and consumption[15], mandatory menu labelling has the potential to effect change in the nutritional content of food eaten from out of home sources. Implementation of mandatory menu labelling is required before more robust longitudinal evidence of effect can be generated.

Alongside menu labelling, other strategies are likely to be required to improve the energy and nutritional content of food sourced out of home. This may include strategies to address availability, affordability and marketing; as well as those to provide individuals with the skills and information required to make ‘healthier’ choices. Further research is required to understand the most effective, efficient and equitable combination of strategies.

## Conclusion

Popular UK restaurant chains which provided voluntary menu labelling served items with less fat and salt than those without such labelling. Mandatory menu labelling has the potential to improve the nutritional profile of food served out of home. Some menu items from restaurants both with and without menu labelling had very large portion sizes, and were nutritional imbalanced. Further work is required to establish the most effective, efficient and equitable strategies to improve the nutritional profile of food served out of home.

## Supporting information

S1 Table Distribution of energy and nutrients in 9605 menu items from 42 popular UK chain restaurants, stratified by food category. (DOCX)

S2 Table Summary of linear regression models comparing energy and nutritional content of 9605 menu items from 42 popular UK restaurants with and without in-store menu labelling, stratified by food category. (DOCX)

S1 File Dataset. (XLSX)
