# Correction: Differences in energy and nutritional content of menu items served by popular UK chain restaurants with versus without voluntary menu labelling: A cross-sectional study

**DOI:** 10.1371/journal.pone.0226704

**Published:** 2019-12-12

**Authors:** Dolly R. Z. Theis, Jean Adams

The number of restaurants stated to have menu labelling in store is incorrect throughout the article. The correct number of restaurants that have menu labelling in store is 14 (33%). In light of this error, the following updates should be noted:

- In the Methods and findings subsection of the Abstract, there is an error in the fifth sentence. The correct sentence is: Of these, 14 (33%) voluntarily provided menu labelling.
- In the Results, there is an error in the second sentence of the second paragraph. The correct sentence is: Of the 42 included restaurants with online nutritional information, 14 (33%) voluntarily provided menu labelling.
- In the Results, there are errors in the third sentence of the second paragraph. The correct sentence is: Twelve of the 14 restaurants that provided voluntary menu labelling were in the top 50 by sales in 2013; 38 of the 55 functioning restaurants with functioning websites that voluntarily provided neither menu labelling or online nutritional information were in the bottom 50 by sales in 2013.
- In the Discussion, there is an error in the second sentence of the second paragraph. The correct sentence is: Of 100 restaurant chains considered, 42 provided energy and nutritional information online, of which 14 provided any of this information on the restaurant menu.
- In the Interpretation of findings subsection of the Discussion, there is an error in the fifth sentence of the third paragraph. The correct sentence is: It is notable that 12 of the 14 restaurants that provided voluntary menu labelling were in the top 50 by sales.

There is an error in Table 2. ‘Domino’s Pizza’ (ranked 6) should be colored orange instead of green.

**Table 2:** Total UK Sales and UK units in 2013, presence of online nutritional information, and voluntary menu labelling in 100 popular UK chain restaurants.

Rank	Restaurant Name	2013 UK Sales (£000)*	2013 UK Units*	Online energy/nutritional information	Voluntary menu labelling
1	McDonald’s	£1,810,000	1,222	Yes	Yes
2	Wetherspoon	1,217,000	905	Yes	Yes
3	Costa Coffee	937,000	1,755	Yes	Yes
4	Greggs	787,000	1,671	Yes	Yes
5	KFC	684,500	850	Yes	Yes
6	Domino’s Pizza	622,500	771	Yes	No
7	Starbucks	606,000	764	Yes	Yes
8	Pizza Hut	532,000	685	Yes	No
9	Subway	531,000	1,590	Yes	Yes
10	Nando’s	455,000	290	Yes	No
11	PizzaExpress	411,000	421	Yes	No
12	Burger King	383,000	484	Yes	Yes
13	Pret A Manger	319,000	270	Yes	Yes (food only)
14	Vintage Inns	307,000	193	No	No
15	Caffe Nero	305,000	550	Yes	Yes (food only)
16	Frankie & Benny’s	207,000	209	No	No
17	Harvester Salad & Grill	196,000	210	No	No
18	Wagamama	179,000	105	Yes	No
19	Sizzling Pubs	174,000	220	No	No
20	Ember Inns	170,000	130	No	No
21	Brewers Fayre	163,000	145	Yes	No
22	Hungry Horse	161,000	199	No	No
23	T.G.I Friday’s	153,000	65	No	No
24	Beefeater Grill	146,000	140	Yes	No
25	Prezzo	136,000	194	No	No
26	Chef & Brewer Pub Co.	125,000	135	Yes	No
27	Crown Carveries	123,000	114	No	No
28	Table Table	116,000	105	Yes	No
29	Taylor Walker	112,000	113	Yes	No
30	Toby Carvery	112,000	154	Yes	No
31	Revolution Vodka Bars	109,000	67	No	No
32	Zizzi	109,000	130	Yes	No
33	Carluccio’s	104,000	76	No	No
34	Jamie’s Italian	102,000	37	Yes	No
35	EAT	99,000	112	Yes	Yes (food only)
36	Nicholson’s	99,000	77	No	No
37	ASK	95,000	110	Yes	No
38	Fayre & Square	95,000	157	Yes	No
39	The Slug and Lettuce	95,000	73	No	No
40	Café Rouge	87,000	127	No	No
41	Papa John’s	86,000	246	Yes	No
42	Yate’s	84,000	69	Yes	No
43	Sayers the Better Bakers	78,000	178	No	No
44	YO! Sushi	75,000	66	Yes	Yes
45	All Bar One	73,000	47	Yes	No
46	Ben & Jerry’s	72,000	265	Yes	No
47	Bella Italia	66,000	91	No	No
48	Strada	63,500	68	No	No
49	Chicken Cottage	61,000	129	Yes	No
50	John Barras	60,200	126	Yes	No
51	Chiquito	54,000	70	No	No
52	Gaucho Grill	53,200	16	No	No
53	Patisserie Valerie	53,000	108	No	No
54	Old English Inns	52,500	55	Yes	No
55	O’Neill’s	49,000	49	No	No
56	Scream	48,600	43	No longer exists	NA
57	Gourmet Burger Kitchen	46,200	60	Yes	No
58	Davy’s	45,100	28	No	No
59	Flaming Grill Pub Co.	42,000	87	Yes	No
60	Loch Fyne	42,000	42	No	No
61	Browns Bar & Brasserie	38,500	27	No	No
62	Giraffe	38,200	50	No	No
63	Brasserie Blanc	38,000	19	No	No
64	La Tasca	37,000	38	No	No
65	Cote Restaurants	36,700	46	No	No
66	Miller & Carter	35,000	29	No	No
67	Wildwood Restaurants	33,100	18	No	No
68	Wacky Warehouse	32,200	75	No	No
69	Hollywood Bowl	32,000	46	No	No
70	Favourite Fried Chicken	31,800	85	No	No
71	Pitcher & Piano	31,700	18	No	No
72	Byron	31,000	34	No	No
73	Meet & Eat Pub & Grill	31,000	38	No	No
74	Piccolino Ristorante e Bar	30,500	21	No	No
75	PAUL	30,400	31	Yes	No
76	Le Pain Quotidien	30,000	24	No	No
77	Las Iguanas	29,800	34	No	No
78	Little Chef	29,500	78	No	No
79	Loungers	29,500	38	No longer exists	NA
80	Cosmo	28,100	15	No	No
81	Handmade Burger Co.	27,300	18	No	No
82	San Carlo	27,000	13	No	No
83	Jamies Wine Bars	26,000	10	No	No
84	Wimpy	26,000	110	Yes	Yes
85	Ed’s Easy Diner	25,700	23	No	No
86	Pizza GoGo	25,700	95	No	No
87	Krispy Kreme	25,400	52	Yes	No
88	Bill’s	25,200	31	Yes	No
89	Busaba Eathai	25,200	10	No	No
90	Pizza Kitchen & Bar	25,200	24	Website invalid	No
91	Gusto	24,800	10	No	No
92	Muffin Break	23,300	51	No	No
93	Walkabout	23,200	27	Yes	No
94	Baguette Express	23,000	70	No	No
95	Chimichanga	22,800	37	No	No
96	AMT Coffee Bars	22,600	60	No	No
97	Dixy Chicken	22,300	82	No	No
98	Itsu	21,700	43	Yes	Yes
99	The Restaurant Bar & Grill	21,500	11	No	No
100	Aagrah	21,400	16	No	No

Green: restaurants with nutritional information available online and voluntary menu labelling

Orange: restaurants with nutritional information available online, but no voluntary menu labelling

Red: restaurants with no nutritional information available online, and no voluntary menu labelling

Unshaded: restaurant no longer existed at the time of data collection

* Based on Technomic’s 2013 list.
