# Supplementary material for: Differences in energy and nutritional content of menu items served by popular UK chain restaurants with versus without voluntary menu labelling: A cross-sectional study
Source: PLoS One. 2019 Oct 16;14(10):e0222773. doi: 10.1371/journal.pone.0222773 (PMC6795485; doi:10.1371/journal.pone.0222773)
Supplement: S1 Table — (DOCX) [file pone.0222773.s001.docx]

**S1 Table. Distribution of energy and nutrients in 9605 menu items from 42 popular UK chain restaurants, stratified by food category**

| Food category | Nutrient | Median (25^th^ – 75^th^ centile) | Min - max | *DRI |
| --- | --- | --- | --- | --- |
| Appetisers & sides (n=1303) | Energy (kcal) | 158 (94 – 338) | 3 – 1987 | 2000 |
|  | Fat (g) | 6.9 (1 – 18.1) | 0 – 138.28 | <70g |
|  | Saturated Fat (g) | 1.8 (0.1 – 4.5) | 0 - 45 | <20g |
|  | Carbohydrates (g) | 17.8 (8.8 – 30.3) | 0 – 184.4 | At least 260g |
|  | Sugar (g) | 3.4 (1.8 – 5.8) | 0 – 74.8 | 90g |
|  | Protein (g) | 5 (2.8 – 11.5) | 0 – 164.6 | 50g |
|  | Salt (g) | 0.51 (0.1 – 1.4) | 0 - 29 | 6g |
| Baked goods (n=509) | Energy (kcal) | 267 (203 – 361) | 21 – 838 | 2000 |
|  | Fat (g) | 7.5 (4.1 – 16) | 0 – 44.4 | <70g |
|  | Saturated Fat (g) | 3.9 (1.4 – 6.4) | 0 – 22.9 | <20g |
|  | Carbohydrates (g) | 42 (27.7 – 53.5) | 0 – 100 | At least 260g |
|  | Sugar (g) | 3.7 (2.1 – 12.5) | 0 – 54 | 90g |
|  | Protein (g) | 6.7 (4.8 – 9.6) | 0 – 36.1 | 50g |
|  | Salt (g) | 0.74 (0.5 – 1.14) | 0 - 7 | 6g |
| Beverages (n=1929) | Energy (kcal) | 173 (103 – 261) | 1 – 2129 | 2000 |
|  | Fat (g) | 5.4 (1.5 – 10.3) | 0 – 111.6 | <70g |
|  | Saturated Fat (g) | 2.6 (0.3 – 6.5) | 0 – 55 | <20g |
|  | Carbohydrates (g) | 25.1 (13 – 38.15) | 0 – 253.4 | At least 260g |
|  | Sugar (g) | 22.6 (11.4 – 33.95) | 0 – 212.7 | 90g |
|  | Protein (g) | 4.3 (1.6 – 8.1) | 0 – 31.6 | 50g |
|  | Salt (g) | 0.2 (0.1 – 0.32) | 0 – 2.8 | 6g |
| Burgers (n=350) | Energy (kcal) | 854.5 (554.75 – 1349.25) | 74 – 5961 | 2000 |
|  | Fat (g) | 42.6 (25.25 – 71.98) | 1.1 – 412 | <70g |
|  | Saturated Fat (g) | 12.95 (6 – 22.38) | 0.1 – 162.2 | <20g |
|  | Carbohydrates (g) | 67.7 (51.5 – 117.03) | 0.1 – 378.8 | At least 260g |
|  | Sugar (g) | 11.1 (8.28 – 16) | 0 – 81.7 | 90g |
|  | Protein (g) | 37.4 (25.65 – 55.13) | 3.5 – 212.6 | 50g |
|  | Salt (g) | 3.3 (2.1 – 4.47) | 0.2 - 29 | 6g |
| Desserts (n=666) | Energy (kcal) | 334.5 (187.5 – 517.5) | 5 – 2244 | 2000 |
|  | Fat (g) | 14.35 (7.4 – 23.9) | 0 – 92.5 | <70g |
|  | Saturated Fat (g) | 7.1 (4.1 – 11.73) | 0 – 46 | <20g |
|  | Carbohydrates (g) | 42.45 (23.28 – 66.63) | 1.3 – 262.4 | At least 260g |
|  | Sugar (g) | 29.45 (19.2 – 48.25) | 0 – 228.3 | 90g |
|  | Protein (g) | 4.8 (2.5 – 6.9) | 0 – 46 | 50g |
|  | Salt (g) | 0.21 (0.1 – 0.5) | 0 - 29 | 6g |
| Fried potatoes (n=238) | Energy (kcal) | 410.5 (309 – 546) | 73 – 1422 | 2000 |
|  | Fat (g) | 19.3 (14.7 – 29.4) | 0.9 – 78.6 | <70g |
|  | Saturated Fat (g) | 2.2 (1.7 – 5.6) | 0.3 – 29.8 | <20g |
|  | Carbohydrates (g) | 51.2 (37.8 – 65.1) | 0.7 – 179.2 | At least 260g |
|  | Sugar (g) | 1.65 (0.9 – 4.2) | 0 – 53.1 | 90g |
|  | Protein (g) | 5.3 (3.6 – 8.4) | 0.8 – 43.1 | 50g |
|  | Salt (g) | 0.5 (0.17 – 1.21) | 0 - 8 | 6g |
| Mains (n=1196) | Energy (kcal) | 686 (415.25 – 970.75) | 52 – 2731 | 2000 |
|  | Fat (g) | 30.7 (16.5 – 49.85) | 0 – 190.2 | <70g |
|  | Saturated Fat (g) | 8.2 (3.5 – 15.6) | 0 – 72.8 | <20g |
|  | Carbohydrates (g) | 61 (32.48 – 93) | 0 – 216.3 | At least 260g |
|  | Sugar (g) | 7.9 (4.03 – 14.08) | 0 – 122.2 | 90g |
|  | Protein (g) | 32.8 (19.43 – 51.6) | 0.2 – 165.2 | 50g |
|  | Salt (g) | 2.5 (1.4 – 3.7) | 0 – 13.5 | 6g |
| Pizza (n=1444) | Energy (kcal) | 645 (507.25 – 825) | 170 – 1602 | 2000 |
|  | Fat (g) | 23.4 (17.4 – 32.78) | 1.1 –76.8 | <70g |
|  | Saturated Fat (g) | 10 (7.2 – 13.8) | 0 – 46.5 | <20g |
|  | Carbohydrates (g) | 74.7 (58.8 – 91.95) | 15.2 – 163.2 | At least 260g |
|  | Sugar (g) | 13.6 (10.8 – 16.8) | 0 – 42.6 | 90g |
|  | Protein (g) | 30.3 (23.7 – 38.7) | 0 – 97.2 | 50g |
|  | Salt (g) | 3.6 (2.8 – 4.56) | 0.3 – 8.88 | 6g |
| Salads (n=271) | Energy (kcal) | 301 (82 – 466) | 7 – 1160 | 2000 |
|  | Fat (g) | 15.1 (3.7 – 24.7) | 0 – 71.1 | <70g |
|  | Saturated Fat (g) | 2.3 (0.9 – 5.6) | 0 – 33 | <20g |
|  | Carbohydrates (g) | 13.6 (4.7 – 33.4) | 1 – 112.1 | At least 260g |
|  | Sugar (g) | 5 (3 – 9) | 1 – 33 | 90g |
|  | Protein (g) | 15 (1.7 – 29.8) | 0.2 – 68.1 | 50g |
|  | Salt (g) | 1.2 (0.3 – 2.27) | 0 – 6.2 | 6g |
| Sandwiches (n=648) | Energy (kcal) | 480 (396.5 – 586) | 89 – 4540 | 2000 |
|  | Fat (g) | 19.4 (13.5 – 26.2) | 0.7 – 241.1 | <70g |
|  | Saturated Fat (g) | 5.65 (2.8 – 9.1) | 0.1 – 76.5 | <20g |
|  | Carbohydrates (g) | 48.55 (39.33 – 61) | 0 – 424.7 | At least 260g |
|  | Sugar (g) | 5.4 (3.2 – 9.7) | 0 – 72.8 | 90g |
|  | Protein (g) | 22.8 (17 – 32.1) | 2.5 – 146.2 | 50g |
|  | Salt (g) | 2.4 (1.7 – 3.2) | 0 – 17.71 | 6g |
| Soup (n=186) | Energy (kcal) | 260.5 (159.25– 348.5) | 32 – 1332 | 2000 |
|  | Fat (g) | 10.3 (4.75 – 15.78) | 0.3 – 60.3 | <70g |
|  | Saturated Fat (g) | 4.2 (1.78 – 9) | 0 – 25 | <20g |
|  | Carbohydrates (g) | 27.35 (14 – 36.4) | 0 – 172.4 | At least 260g |
|  | Sugar (g) | 8.05 (3.9 – 12) | 0 – 36 | 90g |
|  | Protein (g) | 8.35 (5 – 18) | 0 – 74.4 | 50g |
|  | Salt (g) | 2 (1.7 – 3) | 0 – 10.9 | 6g |
| Toppings & ingredients (865) | Energy (kcal) | 90 (48 – 168) | 2 – 1156 | 2000 |
|  | Fat (g) | 4.9 (0.3 – 12.35) | 0 – 79.1 | <70g |
|  | Saturated Fat (g) | 1.4 (0.1 – 4.25) | 0 – 40 | <20g |
|  | Carbohydrates (g) | 4.4 (1.1 – 12) | 0 – 90.7 | At least 260g |
|  | Sugar (g) | 1.6 (0.2 – 5.9) | 0 – 64.8 | 90g |
|  | Protein (g) | 1.5 (0.4 – 6.3) | 0 – 51.8 | 50g |
|  | Salt (g) | 0.4 (0.01 – 0.8) | 0 – 15.01 | 6g |

*DRI: dietary reference intake
