# Supplementary material for: Differences in energy and nutritional content of menu items served by popular UK chain restaurants with versus without voluntary menu labelling: A cross-sectional study
Source: PLoS One. 2019 Oct 16;14(10):e0222773. doi: 10.1371/journal.pone.0222773 (PMC6795485; doi:10.1371/journal.pone.0222773)
Supplement: S2 Table — (DOCX) [file pone.0222773.s002.docx]

**S2 Table. Summary of linear regression models comparing energy and nutritional content of 9605 menu items from 42 popular UK restaurants with and without in-store menu labelling, stratified by food category**

| Food category | Energy/nutrient | Regression coefficient | 95% CI (adjusted for clustering at restaurant level) |
| --- | --- | --- | --- |
| Appetisers & sides (n=1303) | Energy (kcal) | 1.37 | 0.89 to 2.1 |
|  | Fat (g) | 1.45 | 0.78 to 2.67 |
|  | Saturated Fat (g) | 0.95 | 0.59 to 1.5 |
|  | Carbohydrates (g) | 1.29 | 0.89 to 1.85 |
|  | Sugar (g) | 1.15 | 0.77 to 1.72 |
|  | Protein (g) | 1 | 0.62 to 1.67 |
|  | Salt (g) | 1 | 0.47 to 2.17 |
| Baked goods (n=509) | Energy (kcal) | 1.18 | 1.02 to 1.37 |
|  | Fat (g) | 1.74 | 1.26 to 2.39 |
|  | Saturated Fat (g) | 2 | 1.36 to 2.95 |
|  | Carbohydrates (g) | 1.03 | 0.91 to 1.17 |
|  | Sugar (g) | 4 | 2.8 to 5.7 |
|  | Protein (g) | 0.75 | 0.6 to 0.94 |
|  | Salt (g) | 0.57 | 0.45 to 0.72 |
| Beverages (n=1929) | Energy (kcal) | 0.86 | 0.66 to 1.13 |
|  | Fat (g) | 0.79 | 0.47 to 1.36 |
|  | Saturated Fat (g) | 0.86 | 0.39 to 1.9 |
|  | Carbohydrates (g) | 0.86 | 0.63 to 1.18 |
|  | Sugar (g) | 0.89 | 0.63 to 1.26 |
|  | Protein (g) | 0.64 | 0.39 to 1 |
|  | Salt (g) | 0.92 | 0.62 to 1.38 |
| Burgers (n=350) | Energy (kcal) | 0.79 | 0.48 to 1.28 |
|  | Fat (g) | 0.69 | 0.42 to 1.13 |
|  | Saturated Fat (g) | 0.73 | 0.37 to 1.45 |
|  | Carbohydrates (g) | 0.86 | 0.51 to 1.47 |
|  | Sugar (g) | 0.83 | 0.52 to 1.3 |
|  | Protein (g) | 0.97 | 0.63 to 1.49 |
|  | Salt (g) | 0.88 | 0.63 to 1.2 |
| Desserts (n=666) | Energy (kcal) | 1.07 | 0.86 to 1.34 |
|  | Fat (g) | 1.39 | 0.99 to 1.94 |
|  | Saturated Fat (g) | 1.14 | 0.88 to 1.5 |
|  | Carbohydrates (g) | 1 | 0.78 to 1.28 |
|  | Sugar (g) | 0.93 | 0.69 to 1.26 |
|  | Protein (g) | 1.1 | 0.78 to 1.54 |
|  | Salt (g) | 1.05 | 0.67 to 1.62 |
| Fried potatoes (n=238) | Energy (kcal) | 1.08 | 0.59 to 1.98 |
|  | Fat (g) | 1.06 | 0.63 to 1.77 |
|  | Saturated Fat (g) | 0.85 | 0.43 to 1.65 |
|  | Carbohydrates (g) | 1.16 | 0.6 to 2.22 |
|  | Sugar (g) | 0.4 | 0.13 to 1.23 |
|  | Protein (g) | 1.02 | 0.46 to 2.25 |
|  | Salt (g) | 1.53 | 0.44 to 5.4 |
| Mains (n=1196) | Energy (kcal) | 0.91 | 0.63 to 1.32 |
|  | Fat (g) | 0.78 | 0.53 to 1.14 |
|  | Saturated Fat (g) | 0.74 | 0.49 to 1.12 |
|  | Carbohydrates (g) | 1.38 | 0.8 to 2.37 |
|  | Sugar (g) | 0.9 | 0.58 to 1.39 |
|  | Protein (g) | 0.79 | 0.51 to 1.22 |
|  | Salt (g) | 0.88 | 0.59 to 1.33 |
| Pizza (n=1444) | Energy (kcal) | 1 | 0.73 to 1.37 |
|  | Fat (g) | 1.13 | 0.74 to 1.74 |
|  | Saturated Fat (g) | 1.29 | 0.77 to 2.14 |
|  | Carbohydrates (g) | 0.99 | 0.75 to 1.3 |
|  | Sugar (g) | 0.61 | 0.42 to 0.87 |
|  | Protein (g) | 0.96 | 0.75 to 1.24 |
|  | Salt (g) | 0.36 | 0.23 to 0.54 |
| Salads (n=271) | Energy (kcal) | 1.73 | 0.86 to 3.5 |
|  | Fat (g) | 1.46 | 0.84 to 2.52 |
|  | Saturated Fat (g) | 0.81 | 0.55 to 1.2 |
|  | Carbohydrates (g) | 1.54 | 0.82 to 2.9 |
|  | Sugar (g) | 1.28 | 0.87 to 1.9 |
|  | Protein (g) | 1.32 | 0.52 to 3.3 |
|  | Salt (g) | 1.25 | 0.7 to 2.26 |
| Sandwiches (n=648) | Energy (kcal) | 0.87 | 0.76 to 1 |
|  | Fat (g) | 0.82 | 0.66 to 1.02 |
|  | Saturated Fat (g) | 1 | 0.61 to 1.66 |
|  | Carbohydrates (g) | 1.15 | 0.77 to 1.72 |
|  | Sugar (g) | 0.61 | 0.42 to 0.91 |
|  | Protein (g) | 0.77 | 0.64 to 0.92 |
|  | Salt (g) | 0.73 | 0.57 to 0.92 |
| Soup (n=186) | Energy (kcal) | 1.23 | 0.5 to 3.0 |
|  | Fat (g) | 1 | 0.49 to 2.07 |
|  | Saturated Fat (g) | 0.98 | 0.39 to 2.5 |
|  | Carbohydrates (g) | 1.33 | 0.42 to 4.26 |
|  | Sugar (g) | 1.02 | 0.65 to 1.62 |
|  | Protein (g) | 1.38 | 0.59 to 3.19 |
|  | Salt (g) | 1 | 0.6 to 1.79 |
| Toppings & ingredients (865) | Energy (kcal) | 0.67 | 0.4 to 1.1 |
|  | Fat (g) | 0.53 | 0.32 to 0.91 |
|  | Saturated Fat (g) | 0.56 | 0.35 to 0.89 |
|  | Carbohydrates (g) | 0.97 | 0.67 to 1.4 |
|  | Sugar (g) | 1.17 | 0.71 to 1.9 |
|  | Protein (g) | 0.51 | 0.28 to 0.9 |
|  | Salt (g) | 0.68 | 0.32 to 1.5 |
